# *Plasmodium falciparum* population structure inferred by msp1 amplicon sequencing of parasites collected from febrile patients in Kenya

**DOI:** 10.1186/s12936-023-04700-5

**Published:** 2023-09-09

**Authors:** Brian Andika, Victor Mobegi, Kimita Gathii, Josphat Nyataya, Naomi Maina, George Awinda, Beth Mutai, John Waitumbi

**Affiliations:** 1 Basic Science Laboratory, United States Army Medical Research Directorate, Kisumu, Kenya; 2 Department of Biochemistry, Jomo Kenyatta University of Agriculture and Technology, Nairobi, Kenya; 3 Department of Biochemistry, University of Nairobi, Nairobi, Kenya

**Keywords:** Malaria, Multiplicity of infection, *P. falciparum*, *P. falciparummsp1*, Deep sequencing, Genetic diversity

## Abstract

**Background:**

Multiplicity of infection (MOI) is an important measure of *Plasmodium falciparum* diversity, usually derived from the highly polymorphic genes, such as *msp1*, *msp2* and *glurp* as well as microsatellites. Conventional methods of deriving MOI lack fine resolution needed to discriminate minor clones. This study used amplicon sequencing (AmpliSeq) of *P. falciparum msp1 ﻿(Pfmsp1)* to measure spatial and temporal genetic diversity of *P. falciparum*.

**Methods:**

264 *P. falciparum* positive blood samples collected from areas of differing malaria endemicities between 2010 and 2019 were used. *Pfmsp1* gene was amplified and amplicon libraries sequenced on Illumina MiSeq. Sequences were aligned against a reference sequence (NC_004330.2) and clustered to detect fragment length polymorphism and amino acid variations.

**Results:**

Children < 5 years had higher parasitaemia (median = 23.5 ± 5 SD, *p* = 0.03) than the > 5–14 (= 25.3 ± 5 SD), and those > 15 (= 25.1 ± 6 SD). Of the alleles detected, 553 (54.5%) were K1, 250 (24.7%) MAD20 and 211 (20.8%) RO33 that grouped into 19 K1 allelic families (108–270 bp), 14 MAD20 (108–216 bp) and one RO33 (153 bp). AmpliSeq revealed nucleotide polymorphisms in alleles that had similar sizes, thus increasing the K1 to 104, 58 for MAD20 and 14 for RO33. By AmpliSeq, the mean MOI was 4.8 (± 0.78, 95% CI) for the malaria endemic Lake Victoria region, 4.4 (± 1.03, 95% CI) for the epidemic prone Kisii Highland and 3.4 (± 0.62, 95% CI) for the seasonal malaria Semi-Arid region. MOI decreased with age: 4.5 (± 0.76, 95% CI) for children < 5 years, compared to 3.9 (± 0.70, 95% CI) for ages 5 to 14 and 2.7 (± 0.90, 95% CI) for those > 15. Females’ MOI (4.2 ± 0.66, 95% CI) was not different from males 4.0 (± 0.61, 95% CI). In all regions, the number of alleles were high in the 2014–2015 period, more so in the Lake Victoria and the seasonal transmission arid regions.

**Conclusion:**

These findings highlight the added advantages of AmpliSeq in haplotype discrimination and the associated improvement in unravelling complexity of *P. falciparum* population structure.

**Supplementary Information:**

The online version contains supplementary material available at 10.1186/s12936-023-04700-5.

## Background

Malaria is a life-threatening infectious disease caused by parasites of the genus *Plasmodium* transmitted through bites of infected female *Anopheles* mosquitoes. From 2000 to 2016, the World Health Organization (WHO) recorded significant progress in combating malaria in endemic areas. However, data from the 2021 WHO World Malaria Report showed that the progress in reducing global malaria cases is stalling. Malaria cases numbered 241 million, up from 227 million in 2020, 627 000 people died of malaria of which 80% were children younger than 5 years and Africa had 95% of global malaria cases [1]. To combat the disease burden, intensive intervention efforts were put in place, including treatment with anti-malarial combination therapies, use of insecticide-treated bed nets (ITNs) and indoor residual spraying (IRS). In Kenya, malaria is a leading cause of morbidity and mortality with over 80–90% of malaria infections due to *P. falciparum* [2]. The Ministry of Health estimates that 70% of the population in Kenya live in areas where malaria transmission occurs 8–12 months of the year [2].

Early molecular studies revealed that the parasites exist as a pool of genetic “clones” within a single host, and such multi-clones contributes to the ability of *P. falciparum* to evade the host immune response and develop resistance to anti-malarial drugs [3–5]. It has been suggested that multiclonal malaria infections can influence clinical outcomes in a manner that is dependent on transmission intensity [6], and may negatively impact an individual’s response to anti-malarial drug treatment [7]. RTS,S/AS01 remains the most advanced malaria vaccine and is now recommended by WHO as additional armament to help control malaria in children living in regions with moderate to high transmission [1].

MSP1 is the most abundant surface antigen in the blood stage of *P. falciparum* and plays a crucial role in the initial low affinity attachment of parasite to red blood cell membrane during erythrocyte invasion [8]. MSP1 contains 17 blocks of which block 2 shows extensive allelic polymorphism [9, 10], represented by three allelic families namely K1, MAD20 and RO33.

For malaria research, the term MOI (multiplicity of infection) is defined as the number of distinct clones per individual infection. In a given population, the calculated average MOI from the individuals values, has been proposed as a valuable metric for studying infection dynamics, including of transmission intensity [11], and therefore could be used for monitoring success of malaria control programs. Conversely, other studies have demonstrated a lack of correlation between malaria transmission intensity and MOI [12, 13]. One potential confounder in these association studies is the use of different genotyping methods, some of them lacking fine resolution needed to discriminate minor clones. One way of addressing this gap is by use of next generation sequencing of target microsatellites [14].

In this study, *P. falciparum* *msp1* (*Pfmsp1*) was used to illustrate the utility of amplicon deep sequencing (AmpliSeq) in determining the malaria parasite clonal diversity beyond what can be provided by conventional approaches that use allele sizes. SeekDeep, a bioinformatics pipeline designed for analysis of haplotype frequency from amplicon deep sequencing data has been used successfully in several studies investigating malaria population genetics [8, 15, 16].

## Methods

### Sample collection, assay validation and quality control

A laboratory strain of *P. falciparum* 3D7 was used to initiate and maintain a malaria culture as described by [17], with minor modifications. Briefly, growth of the 3D7 parasites was initiated in washed group O^+^ human RBC diluted to 5% haematocrit in complete RPMI 1640 media supplemented with 0.2% bicarbonate, 25 mM HEPES, 50 µg/mL gentamicin and 10% heat inactivated human serum. Culture was maintained in 25 cm^2^ corning flasks (Corning incorporated, Corning NY, USA) with daily replacement of growth medium. To enrich for early ring stages parasites, culture was synchronized with 5% D-sorbitol in distilled water, which lyses RBCs containing late ring stages and other mature parasites [18]. This treatment was repeated every 48 h until > 98% of the parasites were in the early ring stage as confirmed by microscopy. Parasites were allowed to grow to a parasitemia of 3.6% (equivalent to 18,000 parasites/µL). Serial dilutions of the ring stage parasites were made to 0.55 parasites/µL and amplified using a real time qPCR to determine the limit of detection. The lowest parasite density that yielded usable *Pfmsp1* sequence was used as a cutoff for selecting the field samples with adequate parasite density that would good sequence data.

### DNA preparation from *P. falciparum* 3D7 culture and blood samples

QIAamp DNA Blood Mini Kit (Qiagen) was used extract DNA from 200 μl of cultured parasites and study samples as recommended by the manufacturer. A region of *Pfmsp1* (NC_004330.2) from nucleotides 1201627 to 1201710 (includes the K1, MAD20 and RO33 alleles) was amplified in a primary PCR using 5′-CTAGAAGCTTTAGAAGATGCAGTATTG-3′ as forward primer and 5'-CTTAAATAGTATTCTAATTCAAGTGGATCA-3' as reverse primer [19]. A subsequent nested PCR was performed using a combination of degenerate primers (Table 1) to accommodate strain differences in the *Pfmsp1* gene. The primers also included the Illumina adapter overhang. Briefly, in the primary PCR, 3 μl of DNA template, 0.625 µM of each primer and 1X NEB Next HIFI master mix were used in a 25 μl reaction that included initial denaturation at 95 °C for 5 min, followed by 25 cycles of denaturation at 94 °C for 1 min, annealing at 58 °C for 2 min and extension at 72 °C for 2 min, then a single annealing step at 58 °C for 2 min and final extension at 72 °C for 5 min. In the secondary PCR, 2.5 μl of DNA template, 0.2 µM of each primer, 1X NEB Next HIFI master mix were used in a 25 μl reaction. Cycling conditions included initial denaturation at 95 °C for 5 min, followed by 25 cycles of denaturation at 94 °C for 30 s, annealing at 55 °C for 30 s and extension at 72 °C for 30 s, then a final extension at 72 °C for 5 min. Amplicons were visualized on 1% agarose gel stained with gel red (Invitrogen, Carlsbad, CA).

**Table 1:** List of primer mix used for secondary PCR and deep sequencing of *msp1* gene (NC_004330.2)

	Primer sequence	Position
Forward primers mix	5'**TCGTCGGCAGCGTCAGATGTGTATAAGAGACAG**CTAGAAGCTTTAGAAGATGCAGTATTG-3'	1201627
	5'**TCGTCGGCAGCGTCAGATGTGTATAAGAGACAG**NCTAGAAGCTTTAGAAGATGCAGTATTG-3'	
	5'**TCGTCGGCAGCGTCAGATGTGTATAAGAGACAG**NNCTAGAAGCTTTAGAAGATGCAGTATTG-3'	
Reverse primers mix	5'-**GTCTCGTGGGCTCGGAGATGTGTATAAGAGACAG**TGATTGGTTAAATCAAAGAGTTCGG-3'	1202210
	5'-**GTCTCGTGGGCTCGGAGATGTGTATAAGAGACAG**NTGATTGGTTAAATCAAAGAGTTCGG-3'	
	5'-5'-**GTCTCGTGGGCTCGGAGATGTGTATAAGAGACAG**NNTGATTGGTTAAATCAAAGAGTTCGG-3'	
	5'- **GTCTCGTGGGCTCGGAGATGTGTATAAGAGACAG**NNNTGATTGGTTAAATCAAAGAGTTCGG-3'	

“N” represent mixed nucleotides added to accommodate strain differences in the *msp1* gene. Nucleotides in bold face are Illumina adapter overhangs while those in normal face are gene specific primers

### Amplicon library preparation and sequencing

Amplicons were cleaned using AmpureXP beads (Beckman Coulter, IN, USA) followed by a dual indexing PCR to allow multiplexing of samples. For this, a 50 μL reaction consisting of 5 μL of purified amplicons, 5 μL of each Nextera XT i7 and i5 Index Primer (Illumina, USA), 25 μL of NEBNext High-Fidelity 2X PCR Master Mix (New England Bio-labs, MA, US) and 10 μL of PCR grade water (Thermo Fisher Scientific, CA, USA) was made and amplified by PCR at 95 °C for 3 min, followed by 12 cycles of 95 °C for 30 s, 55 °C for 30 s, and 72 °C for 30 s, and a final extension at 72 °C for 5 min. The indexed amplicon libraries were purified with AMPure XP beads according to the manufacturer’s instructions (Beckman Coulter Genomics, CA, USA), and then quantified on Qubit Fluorometer 2.0 using Qubit dsDNA HS assay kit according to the manufacturer’s protocol (ThermoFisher Scientific, CA, USA). Libraries were normalized to a concentration of 4 nM and then pooled. The pooled samples were denatured and diluted to a final concentration of 12 pM, then spiked with 5% PhiX (Illumina, USA) as a sequencing control and then sequenced on MiSeq platform (Illumina, USA) using MiSeq 600 cycle reagent kit V3 (Illumina, USA).

### Haplotype calling and determination of multiplicity of infections

Haplotypes of *Pfmsp1* were determined using SeekDeep v2.6.0 [20]. Briefly, raw sequencing reads were filtered and trimmed based on the read length and quality scores using the *extractor* module in SeekDeep with the paired-end feature. After quality filtering, the reads were merged, chimeras removed and the sequences clustered at the sample level by *qluster*, and finally assembled based on the *msp1* reference gene (NC_004330.2) to generate *msp1* haplotypes. The assembled haplotypes were analyzed by *processCluster* algorithm which compared sample haplotypes and generated individual and population-level haplotypes and statistics. A final mapping of all sequence reads to selected reference sequences was performed with the CLC Genomics workbench (CLC Inc, Aarhus, Denmark) and queried against the nucleotide database (GenBank) using the Nucleotide Basic Local Alignment Search Tool (BLASTn) [21]. A haplotype was defined as a group of sequences within a cluster that represented the same allele of *Pfmsp1*. The MOI, defined as the number of distinct *msp1* haplotypes in an individual infection (varying by length and at the nucleotide level) was determined for the different alleles (K1, MAD20 and RO33). The number of distinct genotypes for the K1, MAD20 and RO33 in each sample were added and the sum regarded as the MOI for that individual. The calculated group average was regarded as mean MOI. The expected heterozygosity was calculated from the frequencies of the different alleles within the population according to the formula: H_e_ = 1–Σ(pi^2), where H_e_ is the expected heterozygosity, pi is the frequency of the i-th allele in the population.

### Statistical analysis

GraphPad prism and R software were used for visualization and statistical analyses. Box plots comparing the identity between groups were created with GraphPad Prism 9 software [22]. Paired sample t-test was used to compare parasite densities (Ct-values) in the different age groups. Sequence tables generated by SeekDeep were analysed using R with the packages Phyloseq v.1.22 [23] along with packages ggpubr v0.2.5 [24] and vegan v.2.5 [25]. Figures were generated using the following R packages: ggplot2 v.3.2.1 [26], ggthemes v.4.2.0 [27], cowplot v.1.0.0 [28] and viridis v.0.5.1 [29]. A p-value of < 0.05 was considered statistically significant.

## Results

### Limit of detection and quality control

18S rRNA qPCR of cultured parasites consistently detected *P. falciparum* to as low as 0.6 parasite/µl. By Amplicon sequencing of *Pfmsp1,* lowest parasite density that yielded usable *Pfmsp1* sequence was 9 parasites/µL.

### Demography and malaria parasitaemia in the study population

**Fig. 1:**
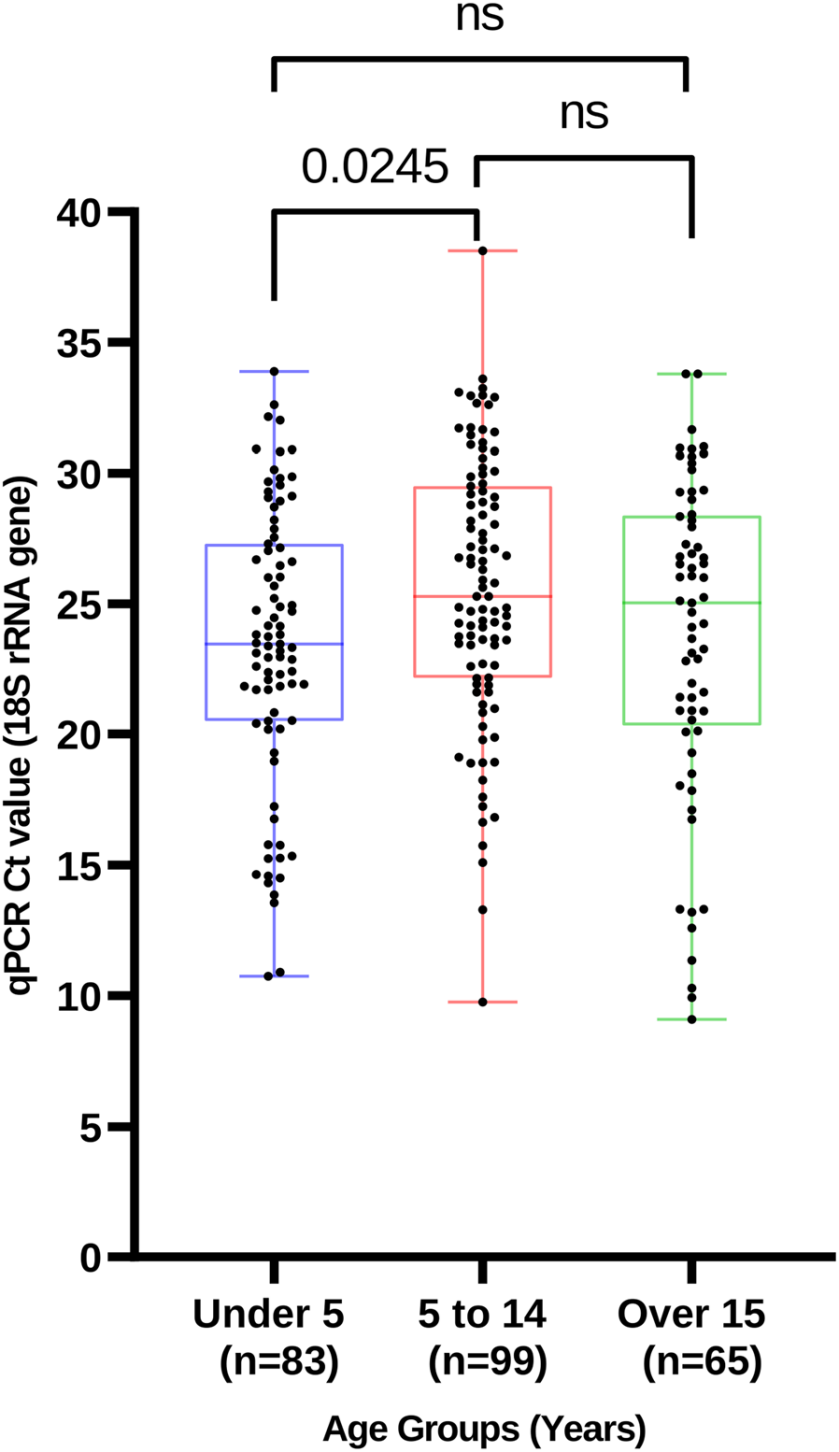
Scatter plots showing qPCR Ct values in different age groups. Children < 5 years had higher parasitaemia than older age groups, but the difference was only significant when compared to the 5–14 years

A total of 247 samples with *P. falciparum* parasitaemia ≥ 9 parasites/µL were selected for inclusion in the study, 122 (49%) were from females, and the median age was 7 years (interquartile range (IQR): 1–66). 83 (30%) were younger than 5 years, 99 (36%) between 5 and 14 years and 92 (34%) > 15 years. A total of 91 (33%) samples were from the malaria endemic lake region, 48 (18%) from the epidemic prone highlands region and 108 (39%) from arid regions that have seasonal malaria. qPCR Ct values were used as surrogate for malaria parasite density (Fig. 1): children < 5 years had higher parasitaemia (mean Ct = 23.37, SD =  ± 5) compared to 5 and 14 years (mean = 25.52, SD =  ± 5) and > 15 years (mean = 23.72 SD =  + 6) years). These differences were only significant for under 5 years and 5 to 14 year age groups (p = 0.0245).

### *Pfmsp1* genetic diversity

#### Genetic diversity by fragment size polymorphisms

**Fig. 2:**
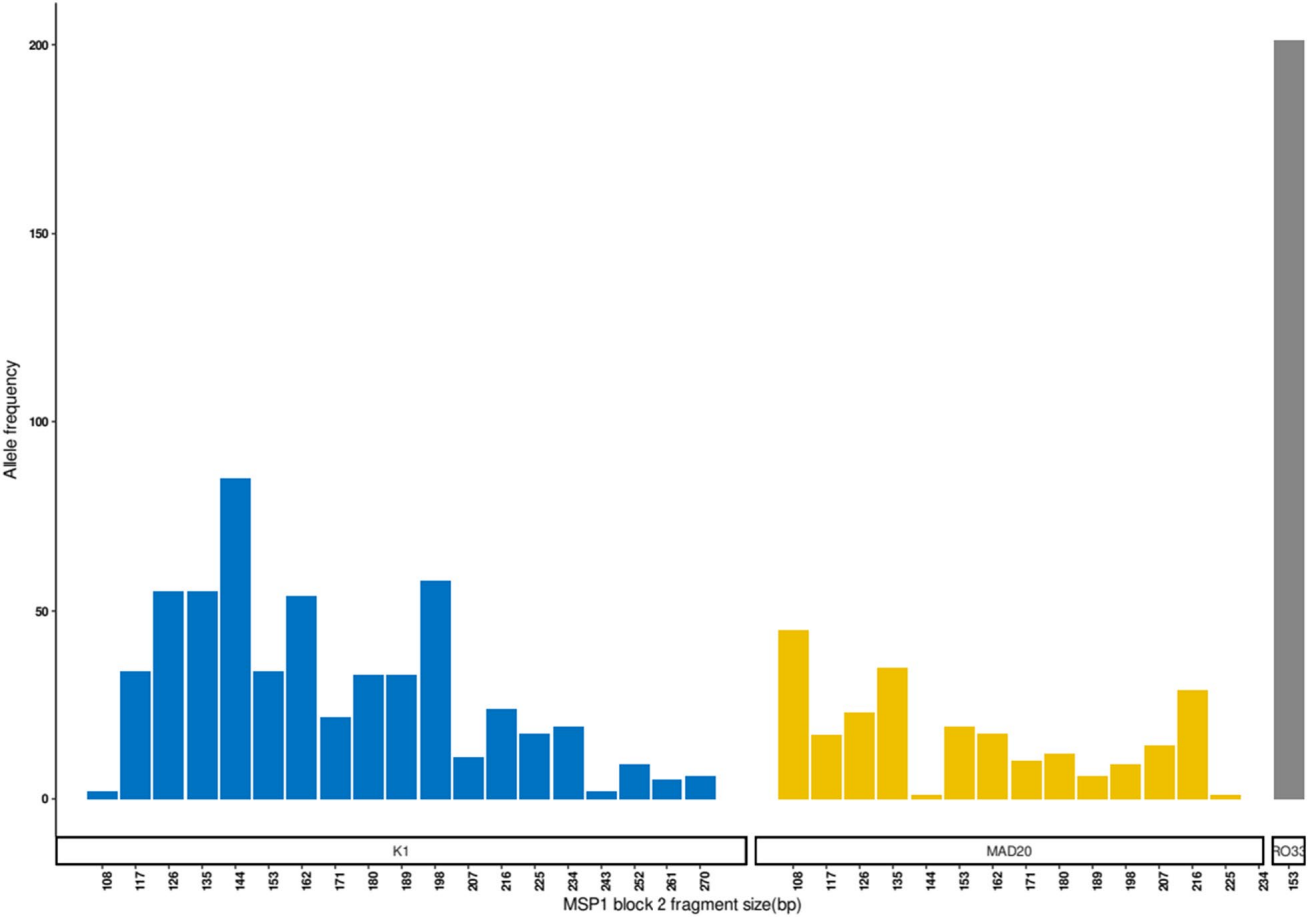
Distribution and prevalence of *Pfmsp1* allelic families in the study samples, based on fragment sizes showing 19 K1 (108–270 bp), 14 MAD20 (108–216 bp) and one RO33 (153 bp)

After quality filtering, the *Pfmsp1* sequences from 274 samples, 247 (84%) passed the Q30 scores. The mean number of reads per sample was 30,998 (range 487–49,983). Based on size, 1,014 alleles (size range: 108–270 bp) were obtained of which, 553 (54.5%) were K1, 250 (24.7%) were MAD20 and 211 (20.8%) were RO33 that grouped into 19 K1 allelic families (108–270 bp), 14 MAD20 (108–216 bp) and one RO33 (153 bp) (Fig. 2).

#### Genetic diversity by nucleotide and amino acids polymorphisms

**Fig. 3:**
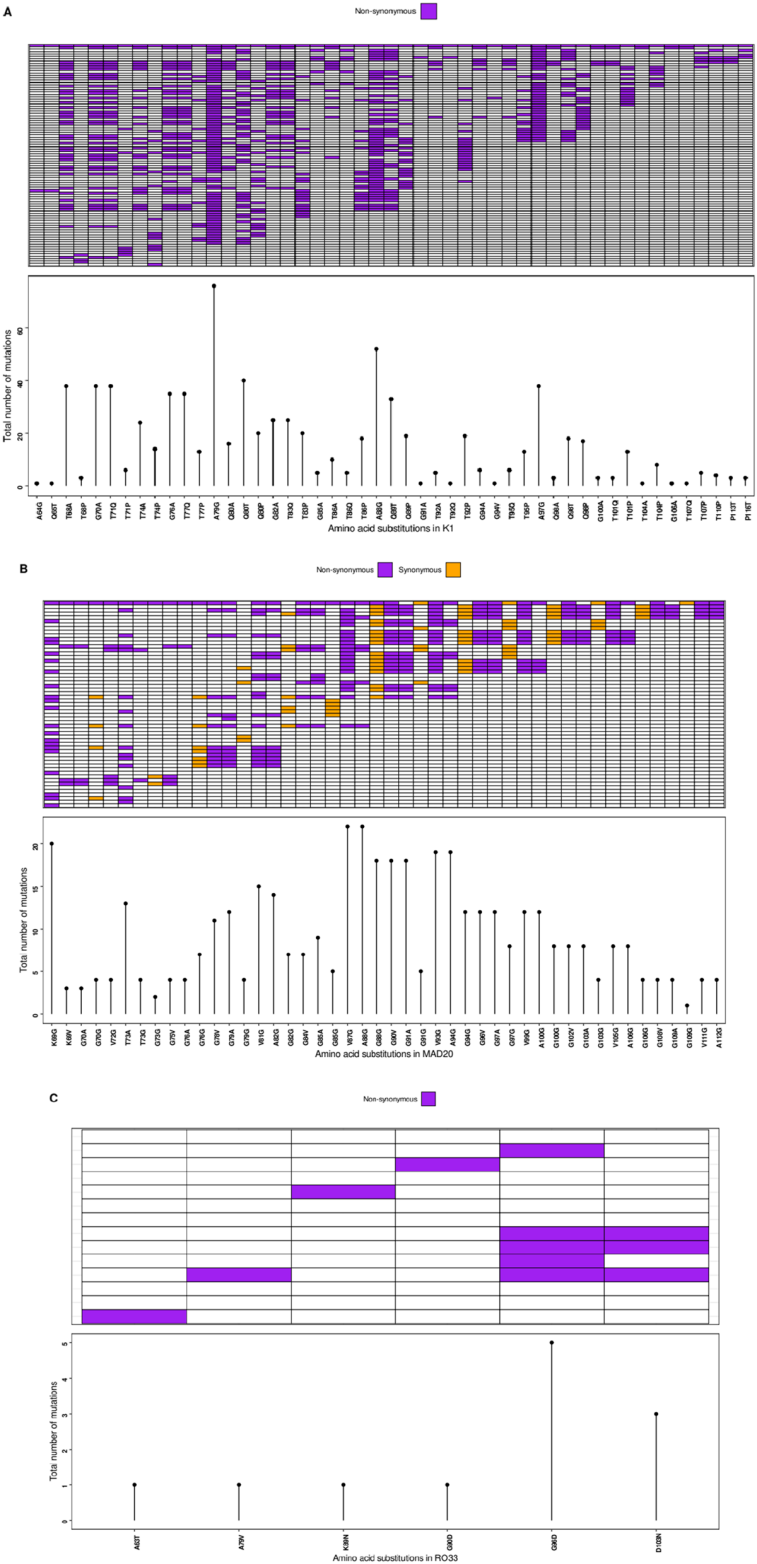
Frequencies of amino acid substitutions across the *Pfmsp1* block 2, showing nonsynonymous amino acid substitutions for K1 and RO33 (Panels A and C) and synonymous and nonsynonymous substitutions for MAD20 (Panel B). The rows represents individual sequences, columns represent the amino acid substitutions. Lollipop plot show the distribution and frequency of the substitutions. For K1, the substitutions were concentrated in the first half of block 2, middle part for MAD20 and last 1/3 for RO33

Sequence analysis of the K1, MAD20 and RO33 alleles revealed nucleotide polymorphisms in alleles that had similar sizes, thus increasing the number of allelic families from 19 to 104 for K1, 14 to 58 for MAD20 and 1 to 14 for RO33 (Additional file 1: Table S1). Most K1 diversity was due to duplications and deletions of the repeat amino acid motifs SGT and SGP and all the 104 sequences of K1 were nonsynonymous (Fig. 3, Panel A). The distribution and frequency of these substitutions were not random and were highest in the first half of block 2. MAD20 sequences were represented essentially by different combinations and deletions of the amino acid motifs SGG, SVA, SVT, and SKG. Synonymous nucleotide replacements were found in the repeat motifs SGG and SVA in 18 out of 58 of MAD20 sequences (Panel B). Unlike K1, the substitutions were concentrated in the middle part. All the 14 substitutions for RO33 were nonsynonymous (Panel C). The 14 RO33 sequences were nearly identical, however six non-synonymous amino acid substitutions were frequently found in codons A63T, A79V, K89N, G90D, G96D and D103N. For RO33, the substitutions were concentrated in the last 1/3 of block 2.﻿

#### Multiplicity of infections

As shown in Table 2, the average number of alleles (shown as MOIs) were relatively stable in all the age groups, but slightly higher in the younger age groups (< 5 years mean = 4.5 ± 0.76, 95% CI) than the 5–14 years (3.9 ± 0.70, 95% CI) and those older > 15 years (2.7 ± 0.90, 95% CI). Females had similar allele frequency to males (mean = 4.2 ± 0.66, 95% CI) compared to males (4.0 ± 0.61, 95% CI). The average number of alleles in the malaria endemic lake region (4.8 ± 0.78, 95% CI) and the epidemic prone highland region (mean = 4.4 ± 1.03, 95% CI) were higher than in the seasonal malaria arid regions (mean = 3.4 ± 0.62, 95% CI). The expected heterozygosity (H_e_), a measure of the probability of being infected by two parasites with different alleles at a given locus in all the regions was high (> 0.98).

**Table 2:** *Plasmodium falciparum* clonal diversity by age, gender and malaria endemicity

	Age bands (years)			Gender		Malaria endemicity		
	< 5	5–14	> 15	Female	Male	Endemic Lake Victoria region	Epidemic prone highlands of Kisii	Seasonal malaria arid regions
Haplotypes	372	389	252	516	497	434	212	367
Mean MOI	4.5	3.9	2.7	4.2	4.0	4.8	4.4	3.4

**Fig. 4:**
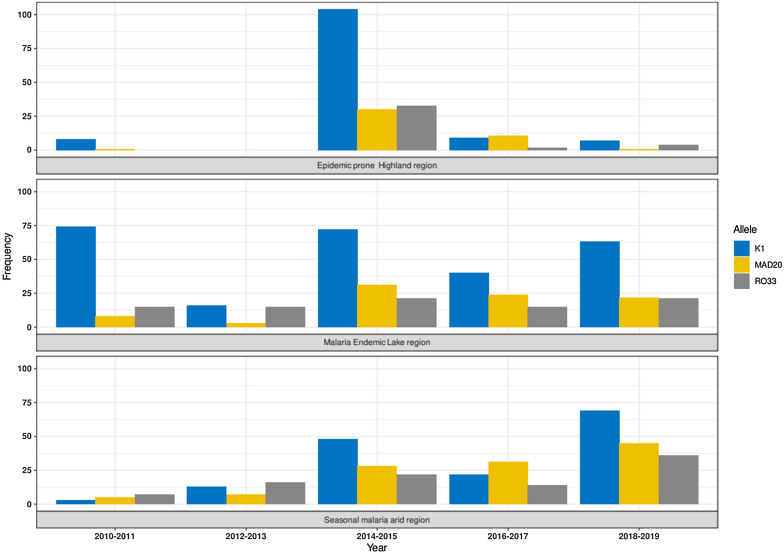
Temporal variation in allelic families in regions of different malaria endemicity. Over time, alleles’ distribution were least stable in the epidemic prone highland region of Kisii, compared to the malaria endemic Lake Victoria region or the seasonal malaria transmission arid region. In general, alleles frequency were low in the 2010–2014 period, and increased thereafter, more so in the Lake Victoria and the seasonal transmission arid regions

In general, the temporal distribution of alleles was least stable in the malaria epidemic prone highland region of Kisii compared to the endemic Lake Victoria region or the seasonal transmission arid region. Overall, alleles frequency were low in the 2010–2014 period, and increased thereafter, more so in the Lake Victoria and the seasonal transmission arid regions (Fig. 4).

## Discussion

In this study, AmpliSeq of the highly polymorphic *Pfmsp1* gene was used to characterize the spatial and temporal allelic structure of *P. falciparum* in three regions of differing malaria endemicities. The choice of *Pfmsp1* was based on several factors. First, it is highly polymorphic, contains several SNPs, likely maintained via balancing selection by immune pressure in the human host and furthermore previous studies in malaria endemic regions have identified over 60 polymorphic sites within *Pfmsp1* [8, 15]. To demonstrate the applicability of AmpliSeq, we first evaluated the lowest parasitaemia density that would give reliable sequence data. 3D7 cultured ring stage malaria parasites produced usable *Pfmsp1* AmpliSeq at a parasitaemia of about 10 parasites/μL. Using this parasitaemia cut-off, 274 samples with *P. falciparum* parasitaemia of ≥ 9 parasites/µL were evaluated. As would be expected, children under 5 years had statistically significant higher malaria parasitaemia compared to those older than 5 years (Fig. 1).

AmpliSeq that combines the size and internal sequence polymorphism improved the power to detect multi clonal infections. As shown in Fig. 2 and Additional file 1: Table S1, *Pfmsp1* AmpliSeq generated 1,014 size alleles that mapped to K1 (54.5%), MAD20 (24.7%) and RO33 (20.8%) and grouped to 34 allelic families (19 K1, 14 MAD20 and one RO33). By including sequence polymorphisms internal to the sequences, the overall increase in the number of allelic families was by 5*x* (34 to 176): 5.5*x*﻿ for K1 (19 to 104), 4.1*x*﻿ for MAD20 (from 14 to 58) and 14*x*﻿ for RO33 (from 1 to 14). Clearly, the use of size to deduce clonal multiplicity underestimates the number of clones in an infection. These findings corroborates previous studies that used AmpliSeq for estimating MOI [30–33]. As has been observed in other studies, K1 was found to be the dominant allelic family [34–36]. This is unlike the RO33 that was reported as the dominant allele in parasites collected from Malaysia [37], Brazil [38], and Gabon [39] and unlike MAD20 allele that was the most prevalent in Myanmar [40, 41], Thailand [41], Iran [42], Pakistan [43], and Colombia [44], Senegal [45]. *Pfmsp1* haplotyping for the population was high, with a an expected heterozygosity value of 0.98.

Both synonymous and nonsynonymous amino acids substitutions were identified across the *Pfmsp1* block 2 (Fig. 3). For K1 and RO33, only nonsynonymous substitutions were identified (Fig. 3, Panels A and C), while for MAD20, both synonymous and nonsynonymous substitutions were identified (Fig. 3, Panel B). The substitutions were not random: For K1, the substitutions were concentrated in the first half of block 2, middle part for MAD20 and last 1/3 for RO33.

Previous studies have shown that most alleles fluctuate significantly over the years and can differ across endemic areas [46, 47]. The present data suggest unequal allelic structure in the three areas of malaria endemicities (Fig. 4). In general, the period before 2010 and up to end of 2013 was marked by lowest allele frequencies, and indirectly malaria prevalence. This period coincided with the introduction, adoption and widespread use of artemisinin-based combination therapy (ACT) following withdrawal of sulfadoxine/sulfalene-pyrimethamine [48]. The epidemic prone Kisii highland region had little or no malaria prior to 2014. This is not surprising considering that the malaria cases seen in Kisii are largely introduced from the neighboring malaria endemic Lake Victoria region and, therefore, if there is low incidences of malaria in the latter region, there will be even fewer malaria cases in Kisii. Thereafter, there was a big burst in multi clonal infections in 2014–2015 in all the sites. At least for Kisii, this bust coincided with an outbreak of what was referred to as highland malaria [49]. For unknown reasons, infections in Kisii declined to near zero by 2019. This is unlike in the malaria endemic Lake Victoria region where the alleles’ distribution were more stable. In the arid region where malaria has seasonal distribution, the allelic structure has been on increase, and by 2018–2019, the distribution resembled the malaria endemic region. Further studies are needed to determine what is behind the stabilization of malaria cases in the arid region and how much of the change is attributable to climate change.

MOI has been found to be high in children compared to adults and increases with transmission intensity [6, 33, 50]. The current study confirm these observations (Table 2): MOI in children < 5 was higher in younger age group (< 5 years mean = 4.5 ± 0.76, 95% CI) than the 5–14 years (3.9 ± 0.70, 95% CI) and those older > 15 years (2.7 ± 0.90, 95% CI). MOI was also influenced by malaria intensity, was higher in samples from malaria endemic Lake Victoria basin and the semi-arid region compared to the epidemic prone highland region of Kisii. The average number of alleles in the malaria endemic lake region (mean = 4.8 ± 0.78, 95% CI) and the epidemic prone highland region (mean = 4.4 ± 1.03, 95% CI) were higher than in the seasonal malaria arid regions (mean = 3.4 ± 0.62, 95% CI). The expected heterozygote (H_e_) was very high (> 0.98) and was independent of transmission pattern. Similar findings have been reported before [51].

## Conclusion

There are over 500 different *Pfmsp1* sequences that are available in public databases. Our study adds 176 distinct allelic sequences to this database. The *Pfmsp1* antigen has been highly studied as a malaria vaccine candidate and to date, data has demonstrated that MSP1 based vaccine protection against clinical malaria is strain-specific and, therefore, a clear understanding of MSP1 diversity is critical to developing an effective malaria vaccine [52]. Haplotype frequency was influenced by age, gender and transmission settings, highlighting the complexity of determinants of *P. falciparum* population structure. One of the limitation of the study is that the sampling was only possible in patients with parasitaemia cutoff of 10 parasites/μL, thus under representing haplotypes from patients with low parasite density, thereby failing to capture the full diversity of haplotypes present in the population. There is probably no way to solve this shortcoming since technology used is not sensitive enough to sequence very low parasite load. Nevertheless, the analytical depth of AmpliSeq give high confidence that the data obtained is robust and provide a credible overview of the *P. falciparum* population structure in the study populations and regions.

### Supplementary Information

**Additional file 1.** *Pfmsp1* block 2 nucleotide sequences showing the KI (sheet 1), MAD20 ﻿(sheet 2) and RO33 ﻿(sheet 3) allelic families, including the repeat motifs found in K1 and MAD20 (shown in bolded blue fonts) and the polymorphic nucleotides (shown in bold red fonts).

## Data Availability

Raw sequence data generated in this study are available from the National Centre for Biotechnology Information (NCBI) Sequence Read Archive (SRA) under BioProject ID: PRJNA983533.
